# Brain network segregation and integration during painful thermal stimulation

**DOI:** 10.1093/cercor/bhab464

**Published:** 2022-01-03

**Authors:** Gránit Kastrati, William H Thompson, Björn Schiffler, Peter Fransson, Karin B Jensen

**Affiliations:** Department of Clinical Neuroscience, Karolinska Institutet, 17176 Stockholm, Sweden; Department of Clinical Neuroscience, Karolinska Institutet, 17176 Stockholm, Sweden; Department of Clinical Neuroscience, Karolinska Institutet, 17176 Stockholm, Sweden; Department of Clinical Neuroscience, Karolinska Institutet, 17176 Stockholm, Sweden; Department of Clinical Neuroscience, Karolinska Institutet, 17176 Stockholm, Sweden

**Keywords:** brain network, fMRI, pain, time-varying functional connectivity

## Abstract

The present study aimed to determine changes in brain network integration/segregation during thermal pain using methods optimized for network connectivity events with high temporal resolution. Participants (*n* = 33) actively judged whether thermal stimuli applied to the volar forearm were painful or not and then rated the warmth/pain intensity after each trial. We show that the temporal evolution of integration/segregation within trials correlates with the subjective ratings of pain. Specifically, the brain shifts from a segregated state to an integrated state when processing painful stimuli. The association with subjective pain ratings occurred at different time points for all networks. However, the degree of association between ratings and integration/segregation vanished for several brain networks when time-varying functional connectivity was measured at lower temporal resolution. Moreover, the increased integration associated with pain is explained to some degree by relative increases in between-network connectivity. Our results highlight the importance of investigating the relationship between pain and brain network connectivity at a single time point scale, since commonly used temporal aggregations of connectivity data may result in that fine-scale changes in network connectivity may go unnoticed. The interplay between integration/segregation reflects shifting demands of information processing between brain networks and this adaptation occurs both for cognitive tasks and nociceptive processing.

## Introduction

The subjective perception of pain is associated with interactions between brain regions involved in nociceptive processing (Jensen et al. 2016) and regions supporting cognition and emotion (Cheng et al. 2018; Geuter et al. 2020). In terms of brain function, it has been recognized that human cognition and emotion require a balance between functional specialization and integration (Tononi et al. 1994; Fair et al. 2007; Cohen and D'Esposito 2016; Shine et al. 2016), although it is unclear how this balance is achieved during processing of pain. To further our understanding of the interplay between networks supporting the experience of pain, neuroimaging data can be modeled with network theory (Zaki et al. 2007; Baliki et al. 2014; Kim et al. 2019). A network-based approach to model brain connectivity offers the potential to go one step beyond simple identification of brain activity by inferring mechanistic properties relating to the flow of information between brain regions (Bertolero and Bassett 2020). Moreover, variables derived from network-based modeling of functional brain imaging data may have the potential to act as biomarkers of brain disease (Kaiser 2013), notably also for chronic pain conditions (Baliki et al. 2008, 2011).

**Figure 1:**
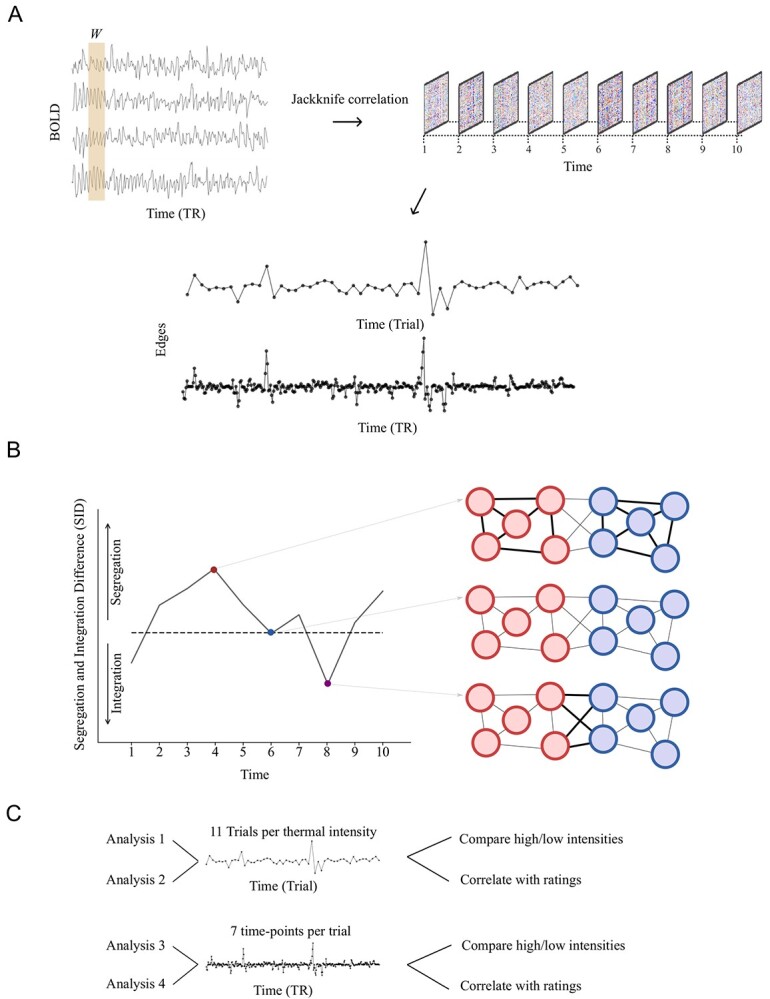
A schematic of our approach to compute brain network segregation and integration. (*A*) BOLD time series were used to compute TVC. TVC was computed both at the level of individual trials and per measured time point (TR = 2.0) using a window-size *W* equal to the length of a trial or to a single time point (using the jack-knife method), respectively. (*B*) The single parameter SID that provides a measure of integration versus segregation is based on estimates of time-resolved within- and between-network degree centrality. (*C*) TVC data were analyzed in four steps. The first two steps considered TVC measured at the trial level and the last two steps treated the data at the single time point level. For each temporal resolution, we compared “high” and “low” thermal corresponding to the two lowest and two highest intensities (analyses 1 and 3). Ratings were given after each trial allowing us to correlate TVC data measured at the trial timescale (analysis 2). Finally, TVC data at the higher resolution were used to investigate degree of correlation between ratings and TVC at different time points within trials (analysis 4).

In contrast to commonly reported “time-aggregated connectivity” data, where results are based on the average from an entire experiment, the subfield of time-varying functional connectivity (TVC) quantifies “fluctuations in functional connectivity” over time (Lurie et al. 2020; see Fig. 1*A*). Previous research has shown that time-varying properties of network integration and segregation can accurately characterize how changes in cognitive processing arise to meet task demand (Cohen and D'Esposito 2016; Shine et al. 2016). This should be of central importance also for pain research as the perception of pain is dynamic and fluctuates even for short periods of time. Moreover, the time dependence of pain perception has implications for the study of chronic pain conditions (Apkarian et al. 2009) as core features of pain pathology are related to cognitive flexibility and neural plasticity, and may not be elucidated when using conventional time-aggregated neuroimaging data, as certain properties may get averaged out.

Today, there is a variety of methods to estimate and quantify TVC, some of which are used to study neural mechanisms related to pain. In a previous study, the relationship between pain and mind wandering (Kucyi et al. 2013) was explored using the sliding window (SW) approach (Allen et al. 2014) and quantified by calculating the standard deviation (SD) for correlated functional magnetic resonance imaging (fMRI) time series. However, our previous findings suggest that SW is not optimal for tracking fast trial-by-trial changes in brain connectivity expected from event-related experimental designs where the sampling rate is low (in the order of 2–3 s for fMRI; Thompson et al. 2018). Typically, SW connectivity is based on long temporal windows (between 40 and 80 s). Aside from the problem of long temporal windows in SW, the use of SDs as quantitative markers of TVC; as used in a previous study of pain; Kucyi et al. 2013) may be problematic, as it captures the underlying static functional connectivity, making a separation between static and time-varying brain connectivity difficult (Thompson and Fransson 2015, 2016).

The aim of the present study was to determine changes in network integration/segregation during thermal pain, using methods optimized for network connectivity events with high temporal resolution.

## Materials and Methods

### Participants

The data set used in the present study was the functional pain neuroimaging data openly available at OpenNeuro portal with accession number ds000140 (https://openneuro.org/datasets/ds000140) (Woo et al. 2018). In brief, a total of 33 right-handed volunteers were included in the study (mean age: 27.9, SD = 9.0, 22 females). Participants reported no history of psychiatric, neurologic, or pain disorders. Detailed information regarding the participants can be found in Woo et al. (2015). Out of the 33 participants, 25 were included in the analysis. Data from two participants were removed due to the presence of excessive movement (framewise displacement [FD] values above 0.5 for more than 20% of the total number of acquired image volumes). Data from six participants were excluded due to mechanical issues for the stimuli equipment or excessive variability in the exact temperature stimuli given.

### Overview

First, we used the jack-knife correlation (JC) method to quantify TVC, which is sensitive to rapid changes in functional connectivity (Richter et al. 2015; Thompson et al. 2018). Second, we estimated the properties of TVC using measures from temporal network theory (Thompson et al. 2017). We estimated the fluctuations of integration and segregation at every time point relative to its average value (Fransson et al. 2018). Thus, in the present study, we combined the JC method with the time-resolved estimates of the degree of network segregation and integration. This strategy effectively provided us with a means to quantify pain-related fluctuations of connectivity that are independent of the static functional connectivity (Fransson and Thompson 2020). The fact that we use time-resolved information about magnitude change in functional connectivity allowed us to associate brain network segregation and integration with subjective pain reports. A schematic description of our approach to assess network segregation and integration is given in Figure 1.

### Experimental Procedures

The experimental protocol that the data set belonged to consisted of a total of nine varying fMRI runs. In our analysis, we used only the five fMRI runs where the participants actively responded after each stimulus. Each run consisted of 11 trials where participants received thermal stimulation and each run consisted of counterbalanced stimuli. The remaining four fMRI runs not considered here employed either manipulated pain sensations with cognitive upregulation or downregulation or investigated contextual effects of increasing each temperature by one step. At each trial, thermal stimulation was delivered to the left inner forearm. During the first 3 s, stimulus intensity was gradually ramped up, followed by a plateau phase lasting for 7.5 s at target temperature. Finally, the stimulus intensity was ramped down during 2 s before being turned off. Thus, the total duration of each trial was 12.5 s and the target temperatures employed were 44.3, 45.3, 46.3, 47.3, and 48.3 °C. In between each trial, participants first rated whether they felt any pain or not. If they felt pain, they rated their pain on a visual analogue scale from 0 (no pain at all) to 100 (worst imaginable pain). If they did not feel any pain, they rated the warmth of the stimuli from 0 (not warm at all) to 100 (very hot). Ratings of warmth were coded from 0 to 100 and ratings of pain were coded as 101–200. See the original paper for more details (Woo et al. 2015).

### fMRI Data Acquisition

Whole-brain fMRI data were acquired on a 3T Philips Achieva TX scanner at Columbia University’s Program for Imaging in Cognitive Science. Structural images were acquired using high-resolution *T*_1_ spoiled gradient recall images for anatomical localization and subsequently warped to a standard space. Functional images were acquired using an echo-planar imaging (EPI) sequence with TR  =  2000 ms, TE  =  20 ms, field of view  =  224 mm, 64 × 64 matrix, 3 × 3 × 3 mm^3^ voxels, 42 interleaved slices, parallel imaging and SENSE factor 1.5. Stimulus presentation and behavioral data acquisition were controlled using E-Prime software (PST Inc.).

### fMRI Data Preprocessing

Data were preprocessed with fMRIPrep (version 20.0.5). A full description of all the preprocessing steps is given in the Supplementary Materials.

### Cleaning of fMRI Data

We used Teneto (v.0.5.2, https://github.com/wiheto/teneto; Thompson et al. 2017) to extract blood-oxygen-level-dependent (BOLD) signal time series from brain regions defined by the Schaefer parcellation atlas (Schaefer et al. 2018) with 400 regions of interest mapped to 7 brain networks (Yeo et al. 2011). Following the parcellation step, data regression of covariates of no interest was done using Teneto (Thompson et al. 2017), implementing tools from Nilearn (version 0.7.0) (Abraham et al. 2014). The confounds that were regressed out included 24 motion parameters, the original 6 motion parameters, first temporal derivatives of the motion parameters, and 12 quadratic terms of the motion parameters and their temporal derivatives. Other regressors include the first six anatomical CompCor components and the global signal, its derivative, and their quadratic terms, FD and hemodynamic response function (Glover) convolved with the events. The latter regressor was removed to reduce the risk of correlations being driven by common stimulus-evoked activation instead of interaction among neural regions (Cole et al. 2019; Lurie et al. 2020). To address the issue of single time points with high motion, fMRI data time series were scrubbed by removing values at time points that exceeded 0.5 in FD and estimating missing values with cubic spline interpolation.

### Deriving Estimates of TVC

We calculated time-varying connectivity estimates at two different temporal scales: 1) single time point estimation and 2) per trial (seven time points). Time points in the BOLD time series corresponding to events were concatenated after data cleaning and scrubbing, and we used the JC approach to calculate estimates of TVC (Richter et al. 2015). Both positive and negative edges of brain connectivity were kept in the analysis. For the analysis at a single time point (*t*) temporal scale, the jack-knife connectivity estimate at *t* was the Pearson correlation when *t* was left out (a leave-one-out estimate). This procedure entails one connectivity estimate for each time point. For the per trial temporal scale analysis, Pearson correlations were calculated but the seven time points associated with the trial were left out (a leave-seven-out estimate). This procedure yielded one estimate per trial (11 per thermal intensity). After JC estimates for both methods, the issue of sign inversion was corrected by multiplying by −1. Furthermore, the variance compression was corrected by scaling each time series to have a mean of 0 and SD of 1. This interpretation means that the “average connectivity” is at 0, and relative increases or decreases throughout in the time series are reflected by the JC estimates. A detailed description of the jack-knife method is given in (Richter et al. 2015; Thompson et al. 2018).

### Estimation of the Segregation Integration Difference between Pairs of Networks

After computing TVC matrices, we applied temporal network measures to compute the degree of segregation and integration between pairs of networks. This summary measure consists of two components, the mean within-network degree centrality and the mean between-network degree centrality (Fransson et al. 2018).

The within-network degree centrality for a given network *G* at time *t* is defined as

$$ {D}_G^t=\frac{2}{N_G\left({N}_G-1\right)}{\sum}_{i,j}^t{A}_{i,j}^t\ i,j\in G,i\ne j, $$

where ${A}_{i,j}^t$ is the connectivity matrix at time point *t* and ${N}_G$ is the number of nodes in network *G*.

The between-network degree centrality at time *t* for networks *G*_1_ and *G*_2_ is defined as:

$$ {D}_{G1,G2}^t=\frac{1}{N_1{N}_2}{\sum}_{i,j}^t{A}_{i,j}^t\ i\in{G}_1,j\in{G}_2, $$

where ${N}_1$ and ${N}_2$ are the number of nodes in *G*_1_ and *G*_2_, respectively.

Once we have computed within- and between-network degree centrality, we can calculate the segregation integration difference (SID) (20) between the two networks *G*_1_ and *G*_2_ at time *t* as:

$$ {\mathrm{SID}}_{G_1,{G}_2}^t=\left(\frac{2}{N_1\left({N}_1-1\right)}{D}_{G_1}^t-\frac{1}{N_2{N}_2}{D}_{G_1,{G}_2}^t\right). $$

Thus, ${\mathrm{SID}}_{G_1,{G}_2}$ is defined by considering the degree centrality within a subset of nodes, which in this case resides in network *G*_1_, and subtract the degree centrality for all edges (connections) that link together nodes in *G*_1_ and *G*_2_. Note that ${\mathrm{SID}}_{G_1,{G}_2}\ne{\mathrm{SID}}_{G_1,{G}_2}$. The global SID for a network *G* is then computed as the average SID across all other networks. ${\mathrm{SID}}_{\mathrm{G}}$ is then used to mean “the segregation of *G* with all other networks with respect to the degree of integration of *G* with all other networks”. If SID for a network *G* at *t* is positive, then *G* displays a higher degree of segregation from all other networks. Conversely, a negative SID value implies that *G* displays a higher degree of integration with all other networks.

### Statistical Significance Testing

For all statistical tests, we compared the two lowest thermal intensities with the two highest thermal intensities. Hence, the third thermal intensity (46.3^o^) was not included in the comparison between low and high thermal intensities. For completeness, see Supplementary Figures 1–3, which displays SID and within- and between-network degree centrality for each thermal intensity.

Statistical test for data averaged over trials was performed with a permutation test implementing *t* test for related samples using permtest_rel function from netneurotools (version 0.2.2). Data were visualized as rain plots implemented in the python package ptitprince (Allen et al. 2019). To test for statistical differences in brain connectivity between high and low thermal intensities at the single time point scale, a permutation test with cluster-level inference with threshold-free cluster enhancement (TFCE) was implemented with MNE tools (version 0.22.0; Gramfort et al. 2014) implementing mne.stats.permutation_cluster_test with one-way analysis of variance. There were 550 observations (number of subjects, *n* = 25 and number of trials, *n* = 22) for low and the same for high thermal intensities. We tested for differences between thermal intensities for seven time points from onset to offset of stimuli. TFCE was used with 10 000 permutations, with steps of 0.2 starting from zero and default TFCE parameters (Smith and Nichols 2009) for height (*h* = 2) and extent (*e* = 0.5). The test was performed for each brain network separately. Each time point across all brain networks was corrected for multiple comparisons with False-discovery rate (FDR) with the Benjamini–Hochberg procedure (Benjamini and Hochberg 1995). Correction for multiple comparisons was done separately for each measure (SID, within- and between-network degree centrality).

To evaluate the relationship between subjective pain ratings and temporal network measures (SID, within- and between-network degree centrality), a skipped Spearman’s rho was implemented (Pernet et al. 2012; Rousselet and Pernet 2012) with the python package pingouin (Vallat 2018) (version 0.3.8). Skipped Spearman’s correlation coefficient is based on the minimum covariance determinant (MCD) and estimates an association after removing bivariate outliers. That is, the robust center of the data is first computed using the MCD estimator. Then, outliers are identified with the box-plot rule by first orthogonally projecting data points onto lines joining each data point to the robust estimate of location (the middle of the data points). Finally, Spearman’s correlation is computed after removing outliers. *P* values were corrected for multiple comparisons with FDR. We associated subjective pain ratings with temporal network parameters measured both at the trial time-scale and at the single time point scale. For the latter, association with subjective ratings was accomplished by focusing at one time point within trials at a time and *P* values were corrected for both time points and brain networks.

In TVC analysis, preprocessing methods that include global signal regression (GSR) has been shown to be the most effective denoising strategies (Lydon-Staley et al. 2019). However, GSR is known to induce spurious negative correlations (Murphy et al. 2009). For completeness, we reanalyzed all results without GSR. The figures and tables can be found in the Supplementary Materials.

## Results

### Aggregated (Low vs. High Thermal Intensity) TVC

**Figure 2:**
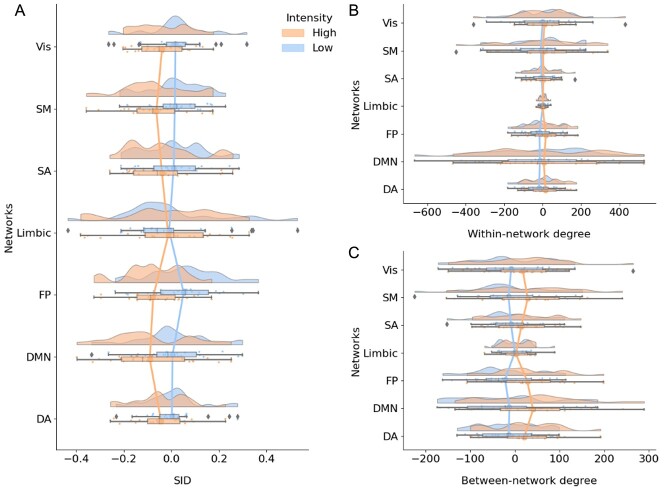
Estimates of SID (*A*), within-network (*B*), and between-network degree centrality (*C*) averaged over all trials. The network measures shown in panels *A*–*C* were computed by aggregating trial data for the two lowest (“low”) and the two highest thermal stimuli intensities (“high”). The vertical lines connect the means of each network.

The differences between high and low thermal intensities were estimated by averaging estimates of network connectivity across all trials of thermal stimulation (see Fig. 2). For six out of seven brain networks, high-temperature trials showed an increased level of network integration, that is, more negative values in the SID parameter as demonstrated in Figure 1, compared to low-temperature trials. The only exception to the general trend toward increased integration during high temperature was found in the limbic network. SID can be driven by a decrease in within-network or an increase in between-network degree centrality. To disentangle the contributions, we quantified within- and between-network centrality separately. We observed a general increase in between-network connectivity following higher thermal intensities (Fig. 2*C*). There was also increased within-network connectivity to some extent. Permutation tests showed that the SID estimate of brain network integration/segregation differed significantly only in the frontoparietal (FP) network when comparing low versus high thermal stimulation, but it did not survive multiple comparisons correction (estimate: −0.128, *P* = 0.02, *P*_corr_ = 0.15). There were no statistically significant differences between low and high thermal intensities in any brain network with regard to within- and between-network degree centrality. Reanalyzing the data without GSR showed that only the default-mode network (DMN) displayed the greatest distinction between high and low thermal intensities, yet this was not statistically significant after correcting for multiple comparisons (Supplementary Fig. 4, Supplementary Tables 7–9).

### The Relationship between TVC for Individual Trials and Ratings of Pain

**Figure 3:**
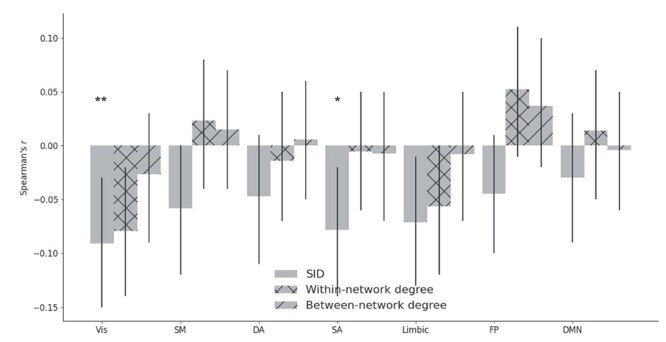
The strength of the statistical relation between temporal network measures (SID, within-, and between-network degree centrality) and pain ratings. Error bars show 95% CI. One star means *P*_corr_ < 0.05 and two stars for *P*_corr_ < 0.01.

Each trial (i.e., the event of a thermal stimulation lasting for seven time points/volumes) was followed by a pain rating and we investigated the relationship between TVC data and subjective ratings of pain during individual trials (see Supplementary Fig. 2 for trial data for each thermal intensity). The degree of association between ratings and each temporal network measure was estimated for each brain network separately and then corrected for multiple comparison (*N* = 1100 [25 participants × 22 trials × 2 levels (low and high intensities)]; degrees of freedom = *N* − 2). There was a statistically significant correlations between ratings and SID values for the visual (Vis), salience (SA), and limbic networks. The results were significant at *P* < 0.05 after correcting for multiple comparisons for the Vis and SA networks (Fig. 3; Vis network: *r* = −0.091, 95% confidence interval [CI] = [−0.15, −0.03], *P* = 0.003, *P*_corr_ = 0.023; limbic network: *r* = −0.071, 95% CI = [−0.13, −0.01], *P* = 0.022, *P*_corr_ = 0.051 and SA network: *r* = −0.078, 95% CI = [−0.14, −0.02], *P* = 0.011, *P*_corr_ = 0.040). See Supplementary Tables 1–3 for detailed results. In Supplementary Figure 5, we show that only the Vis network was statistically significant when analyzing the data without GSR (Supplementary Tables 10–12).

### The Temporal Evolution of TVC Networks Measures Examined at the Level of Single Time Point during Trials of Thermal Stimulation

**Figure 4:**
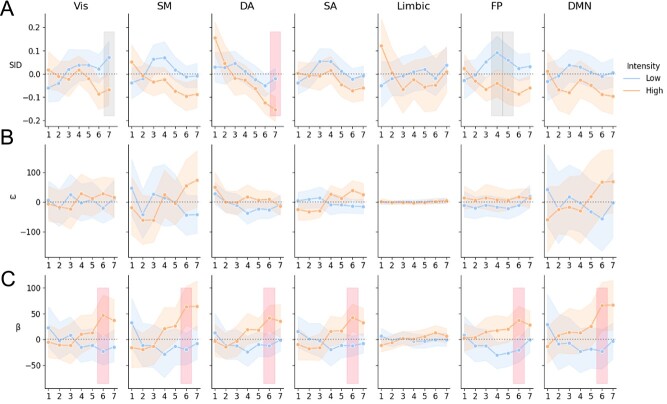
This figure shows SID estimates (*A*), within-network (*B*), and between-network degree centrality (*C*). Shown in *A*–*C* is the average across participants and trials for the seven volumes following thermal stimulation for the two different stimuli (“high” and “low”). Error bars show the 95% CI. The vertical bands represent time points that differed significantly between high and low thermal intensities at *P* < 0.05 (gray bands—corrected for multiple time points, uncorrected for multiple networks, red bands—corrected for multiple time points for multiple networks).

Instead of analyzing TVC as an aggregated measure of data covering an entire trial, this analysis tested if there were any temporal changes of TVC within a trial, consisting of seven time points. The results shown in Figure 4 indicate a higher degree of integration (i.e., lower SID values) during the later phases in high thermal intensity trials. A similar pattern was found for all thermal intensities (see also Supplementary Fig. 3). There was a statistically significant difference in SID for the Vis, dorsal attention (DA), and FP networks; however, only the DA network was significant after correcting for multiple comparisons (Fig. 4*A*) (Vis network: time point = 7, *F* = 1.64, *P* = 0.029, *P*_corr_ = 0.10; DA: time point = 7, *F* = 2.72, *P* = 0.002, *P*_corr_ = 0.017; FP: time points = 3 and 4; *F* = 1.48 and 1.52, *P* = 0.046 and 0.041, *P*_corr_ = 0.32, *P*_corr_ = 0.29). Time-resolved functional connectivity in the somatomotor (SM) network, limbic network, SA network, and DMN did not differ between high and low thermal intensities in terms of segregation and integration. However, there was a statistically significant difference between high and low intensities in terms of between-degree centrality for all networks, with the exception of the limbic network, at the sixth time point (*F* values between 1.56 and 2.52, uncorrected *P* value between 0.0117 and 0.04 and FDR < 0.05).

Analyzing the data without GSR still showed that there is more relative integration for high thermal intensities (Supplementary Fig. 6). There was a statistically significant difference in SID between high and low thermal intensities for the Vis network, SA network, and DMN. However, only the DNM showed statistically significant difference at one time point that survived multiple comparisons (Vis: time point = 6, *F* = 1.72, *P* = 0.024, *P*_corr_ = 0.100; SA: time point = 6, *F* = 1.68, *P* = 0.029, *P*_corr_ = 0.100; DMN: time point = 6, *F* = 2.40, *P* = 0.007, *P*_corr_ = 0.036). There were no statistically significant results for within- and between-network degree centrality.

### Correlations between Time-Varying Connectivity at the Per Time Point Scale and Ratings of Pain

**Figure 5:**
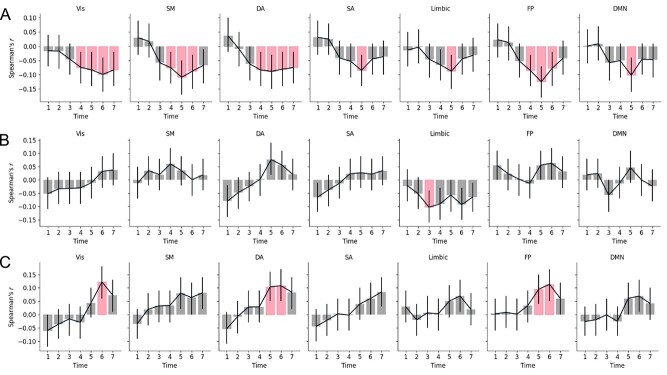
The relationship between temporal network parameters at different time points during trials of thermal stimulation (*A*—SID; *B*—within-network degree centrality; *C*—between-network degree centrality) and subjective ratings of the stimuli. Error bars show the 95% CI. Red bars represent time points with statistically significant correlation (*P* < 0.05, corrected across time points and brain networks).

The statistical significance between network properties and pain ratings at individual image volumes during a trial was analyzed (N = 1100 [25 participants × 22 trials × 2 levels (low and high intensities)]; degrees of freedom = *N* − 2). The results for the SID parameter (Fig. 5*A*) suggest a relatively high degree of network integration with higher pain ratings. All networks displayed small but statistically significant correlation with ratings (*P*_corr_ < 0.05). There were, however, notable differences between time points. For example, the SM, DA, SA, and FP network had positive yet not statistically significant associations for early time points—a finding that suggests a higher relative degree of segregation associated with high pain ratings. From time point four and onwards, this association is reversed, implying increased integration with higher ratings. Figure 5*B* shows that, for all networks except the limbic network, there is a generally increased (positive) within-network degree centrality for higher ratings; however, this was not statistically significant. The limbic network, however, showed one time point where negative within-network degree centrality was associated with higher pain ratings (*r* = −0.103, 95% CI = [−0.16, −0.04], *P* = 0.001, *P*_corr_ = 0.049). This means that with higher ratings, the limbic network displays lesser within-network degree centrality. Figure 5*C* shows that there was a general increase in between-network degree centrality with higher ratings, especially from time point four and onwards. The Vis, DA, and FP network showed time points with statistically significant correlation with pain ratings. Supplementary Tables 4–6 show the detailed results for each metric.

We reran the analysis without GSR (Supplementary Fig. 7). We see the same pattern as with GSR, with more integration for higher pain ratings. However, there were noticeable differences in time points and brain networks. The Vis, DA, SA, FP, and DNM showed statistically significant correlation between SID and pain ratings. Moreover, the between-network degree centrality was statistically significant only for the Vis network. However, the pattern of increased between-network connectivity is still visible, corresponding to time points with increased integration. Supplementary Tables 13–15 show the detailed results.

## Discussion

The ability to track time-varying changes in connectivity between brain networks is of importance for understanding the mechanisms underlying the perception of pain. We quantified the time-varying brain network connectivity and measured the relative degree of segregation and integration for seven canonical networks. Our results showed that brain networks integrate with increased pain. This was observed both at single time point within trials and at time points corresponding to whole trials. However, there were some differences in results between timescales. At the trial timescale, the Vis and SA network showed more integration for higher pain ratings. When investigated at the single time point scale, all brain networks showed increased integration with pain ratings for some time points within trials. These findings illustrate the importance of probing TVC and how aggregating brain network data over time can lead to results being averaged out.

In a recent study, TVC was used to analyze fMRI data acquired during an experiment using a *n*-back working memory task (Fransson et al. 2018). In that study, there was an overall increase in integration between networks during the more demanding 2-back compared to the 0-back task. In this study, we see comparable patterns of increased integration of brain networks during high thermal stimulation. However, in that study, they could single out the FP network, that is highly relevant to the *n*-back task. In the current study, we note a significant increase in integration of several brain networks for higher thermal intensities (Fig. 2). This in line with previous findings of increased co-operation across the brain to support pain perception (Geuter et al. 2020). Since pain has high motivational value and serves as an alarm system for threats to our bodily tissues (Craig 2003), it has the potential to modulate other cognitive and emotional functions. Our findings at the brain network level can be interpreted in terms of increased attention to salient signals to support the multifeatured experience of pain. It is currently unclear if the brain becomes adjusted to persistent pain processing in chronic conditions, and that neural structures associated with attention and cognition become less integrated with time.

The finding of an association between brain integration and pain is in line with earlier findings showing that network modularity decreases with pain (Zheng et al. 2020). Using community detection, Zheng et al. found a large integrated brain network, and reduced between network connectivity to other networks. Here, we used seven canonical resting-state brain networks, and found increased between-network connectivity that may influence the observed integration. This difference is likely due to methodological choices in how brain networks are defined. Furthermore, we unfold temporal fluctuations in functional connectivity and show that the integration/segregation of brain networks only at certain time points within trials capture aspects of the phenomenal experience of pain. For example, note that the integration of the FP and SM networks correlate with ratings for time points 4–6, but this correlation diminishes, while integration of the DA and Vis network continues to correlate with ratings (Fig. 5). We interpret these findings as an increased attentional load associated with high pain levels, in line with the high saliency of intensive pain and need to direct one’s attention to possible dangers (Legrain et al. 2011). It is possible that the DA network is recruited in a dose–response manner to subjective pain intensity and may be an important component for understanding differences in pain perception in addition to traditional “pain networks.” Also, the SA network, limbic network, and DNM show only one time point where integration correlates with pain ratings. Overall, the integration of brain networks could influence the degree to which a stimulus is experienced painful, or not. In contrast to integration, increases in network segregation for lower thermal intensities might reflect baseline values for integration and segregation between resting-state networks. This notion is supported by previous findings of increases in segregation during baseline in between trials (Fransson et al. 2018) and for innocuous stimuli (Zheng et al. 2020).

Most networks, except the limbic network, showed a difference between high and low thermal intensities in terms of between-network degree centrality (Fig. 4*C*) while only the DA network showed a statistically significant difference in terms of SID (Fig. 4*A*). However, SID correlated the most with subjective ratings of pain at the single time point scale (Fig. 5). This suggests that neural correlates of the subjective rating of pain can be distinguished when incorporating the balance between within- and between-network degree centrality, represented in the compound measure of the SID. The full extent of an association between the interplay among networks and subjective ratings of pain may therefore not be evident when SID is decomposed into within- and between-network connectivity.

This study is not without limitations. There is evidence that neural processes associated with pain are highly correlated to saliency (Legrain et al. 2011). Studies that utilize pain stimuli should include salient nonpainful control stimuli (Mouraux et al. 2011) to reveal TVC as it pertains to pain more specifically. Furthermore, putative effects from physiological confounds on the time-varying connectivity properties reported cannot be unconditionally ruled out. For example, even after applying confound regression, correlation between time-varying connectivity estimates still display correlation with nuisance regressors (Nalci et al. 2019). A fluctuating level of arousal has been identified as a possible confound of time-varying connectivity and could potentially act as an influential factor in our analyses (Laumann et al. 2017). However, as argued elsewhere (Lurie et al. 2020), it is likely that cognition and arousal are so heavily intertwined that they should not be attempted to be disentangled from each other (e.g., the subjective experience of pain will likely correspond to levels of arousal).

In TVC analysis, preprocessing methods that include GSR have been shown to be the most effective denoising strategies (Lydon-Staley et al. 2019). However, GSR is known to induce spurious negative correlations (Murphy et al. 2009). For completeness, we reanalyzed all results without GSR. A comparison between the results obtained with and without GSR suggests that removing the global mean influences some brain networks more than others for positive values of the temporal network measures employed here (Supplementary Fig. 8). However, the association between integration and pain was still visible for both analyses yet with differences appearing with respect to brain networks and time points.

The results presented here open up for several new research questions that may deepen our understanding of the brain mechanisms involved in pain perception. For example, it would be interesting to probe specific network nodes and see how they contribute to the integration and segregation of brain networks during the perception of painful stimuli. This avenue of research would add specificity to our results. Furthermore, in this study, we have assumed networks to be static collections of nodes and measured the degree of interaction between networks that is fluctuating across time. Thus, investigating dynamic network membership of the nodes could provide important and detailed information on the functional role of networks during painful conditions and possibly increase our understanding of its role in a more general setting or generalize to other clinically relevant domains such as psychiatric symptoms. Previous findings on static functional connectivity have shown that a large module or subnetwork can be identified during pain with community detection, with higher efficiency within that network than across networks (Zheng et al. 2020). Extending such work to dynamic community detection could reveal how brain networks reconfigure to support pain. Another avenue for further research would be to examine putative behavioral correlates of spontaneous increases and decreases in segregation of brain networks, such as pain catastrophising.

By employing a single time point method to investigate time-varying brain connectivity during different pain intensities, we have shown that network properties vary across brain networks and time, consistent with the changing nature of the subjective experience of pain. During processing of more intense thermal heat stimuli, brain networks shift from a segregated to an integrated state. This is evident both for estimates at the trial timescale and single time point scale. There were some noticeable differences, in that the association between integration and pain ratings was averaged out when TVC was estimated at the trial timescale. We conclude that TVC measured at higher temporal resolution tracks subjective reports more accurately, compared to TVC at a lower resolution. The dynamic interplay between network segregation and integration presumably reflects shifting demands of information processing between networks that are related to the level of thermal stimuli. This adaptation occurs both for cognitive tasks such as working memory and for processing of painful stimuli and could possibly shed light on other clinically relevant brain processes, for example, anxiety.

## Authors’ Contributions

G.K., W.H.T., P.F., and K.J. conceptualized the study. G.K. and W.H.T. analyzed the data. G.K. wrote the first draft. All authors substantially revised the manuscript. All authors contributed to interpretation of results. All authors have read and approved the manuscript.

## Data and Materials Availability

The data can be downloaded at https://openneuro.org/datasets/ds000140. The python package Teneto (https://github.com/wiheto/teneto) was used to compute TVC and SID. All the code used to produce figures and results are available at https://github.com/granitz/twin_pain.

## Supplementary Material

Supplementary_bhab464
