# Supplementary material for: Brain network segregation and integration during painful thermal stimulation
Source: Cereb Cortex. 2022 Jan 3;32(18):4039–49. doi: 10.1093/cercor/bhab464 (PMC9476629; doi:10.1093/cercor/bhab464)
Supplement: Supplementary_bhab464 [file supplementary_bhab464.zip › Supplementary_bhab464.docx]

**Supplementary materials for**

Brain network segregation and integration during painful thermal stimulation

Gránit Kastrati, William Hedley Thompson, Björn Schiffler, Peter Fransson & Karin Jensen

Department of Clinical Neuroscience, Karolinska Institutet, Stockholm, Sweden.

**Content**:

fMRIPrep preprocessing details

Figs. S1 to S8

Table S1 to S15

fMRIPrep references

***fMRI data preprocessing***

Results included in this manuscript come from preprocessing performed using fMRIPrep 20.0.5 (Esteban, Markiewicz, et al. (2018); Esteban, Blair, et al. (2018); RRID:SCR_016216), which is based on Nipype 1.4.2 (Gorgolewski et al. (2011); Gorgolewski et al. (2018); RRID:SCR_002502).

*Anatomical data preprocessing*

The T1-weighted (T1w) image was corrected for intensity non-uniformity (INU) with N4BiasFieldCorrection (Tustison et al. 2010), distributed with ANTs 2.2.0 (Avants et al. 2008, RRID:SCR_004757), and used as T1w-reference throughout the workflow. The T1w-reference was then skull-stripped with a Nipype implementation of the antsBrainExtraction.sh workflow (from ANTs), using OASIS30ANTs as target template. Brain tissue segmentation of cerebrospinal fluid (CSF), white-matter (WM) and gray-matter (GM) was performed on the brain-extracted T1w using fast (FSL 5.0.9, RRID:SCR_002823, Zhang, Brady, and Smith 2001). Volume-based spatial normalization to one standard space (MNI152NLin2009cAsym) was performed through nonlinear registration with antsRegistration (ANTs 2.2.0), using brain-extracted versions of both T1w reference and the T1w template. The following template was selected for spatial normalization: ICBM 152 Nonlinear Asymmetrical template version 2009c [Fonov et al. (2009), RRID:SCR_008796; TemplateFlow ID: MNI152NLin2009cAsym],

*Functional data preprocessing*

For each of the 1 BOLD runs found per subject (across all tasks and sessions), the following preprocessing was performed. First, a reference volume and its skull-stripped version were generated using a custom methodology of fMRIPrep. Susceptibility distortion correction (SDC) was omitted. The BOLD reference was then co-registered to the T1w reference using flirt (FSL 5.0.9, Jenkinson and Smith 2001) with the boundary-based registration (Greve and Fischl 2009) cost-function. Co-registration was configured with nine degrees of freedom to account for distortions remaining in the BOLD reference. Head-motion parameters with respect to the BOLD reference (transformation matrices, and six corresponding rotation and translation parameters) are estimated before any spatiotemporal filtering using mcflirt (FSL 5.0.9, Jenkinson et al. 2002). The BOLD time-series (including slice-timing correction when applied) were resampled onto their original, native space by applying the transforms to correct for head-motion. These resampled BOLD time-series will be referred to as preprocessed BOLD in original space, or just preprocessed BOLD. The BOLD time-series were resampled into standard space, generating a preprocessed BOLD run in MNI152NLin2009cAsym space. First, a reference volume and its skull-stripped version were generated using a custom methodology of fMRIPrep. Several confounding time-series were calculated based on the preprocessed BOLD: framewise displacement (FD), DVARS and three region-wise global signals. FD and DVARS are calculated for each functional run, both using their implementations in Nipype (following the definitions by Power et al. 2014). The three global signals are extracted within the CSF, the WM, and the whole-brain masks. Additionally, a set of physiological regressors were extracted to allow for component-based noise correction (CompCor, Behzadi et al. 2007). Principal components are estimated after high-pass filtering the preprocessed BOLD time-series (using a discrete cosine filter with 128s cut-off) for the two CompCor variants: temporal (tCompCor) and anatomical (aCompCor). tCompCor components are then calculated from the top 5% variable voxels within a mask covering the subcortical regions. This subcortical mask is obtained by heavily eroding the brain mask, which ensures it does not include cortical GM regions. For aCompCor, components are calculated within the intersection of the aforementioned mask and the union of CSF and WM masks calculated in T1w space, after their projection to the native space of each functional run (using the inverse BOLD-to-T1w transformation). Components are also calculated separately within the WM and CSF masks. For each CompCor decomposition, the k components with the largest singular values are retained, such that the retained components’ time series are sufficient to explain 50 percent of variance across the nuisance mask (CSF, WM, combined, or temporal). The remaining components are dropped from consideration. The head-motion estimates calculated in the correction step were also placed within the corresponding confounds file. The confound time series derived from head motion estimates and global signals were expanded with the inclusion of temporal derivatives and quadratic terms for each (Satterthwaite et al. 2013). Frames that exceeded a threshold of 0.5 mm FD or 1.5 standardised DVARS were annotated as motion outliers. All resamplings can be performed with a single interpolation step by composing all the pertinent transformations (i.e. head-motion transform matrices, susceptibility distortion correction when available, and co-registrations to anatomical and output spaces). Gridded (volumetric) resamplings were performed using antsApplyTransforms (ANTs), configured with Lanczos interpolation to minimize the smoothing effects of other kernels (Lanczos 1964). Non-gridded (surface) resamplings were performed using mri_vol2surf (FreeSurfer).

Many internal operations of fMRIPrep use Nilearn 0.6.2 (Abraham et al. 2014, RRID:SCR_001362), mostly within the functional processing workflow. For more details of the pipeline, see the section corresponding to workflows in fMRIPrep’s documentation.

*Copyright Waiver*

The above boilerplate text was automatically generated by fMRIPrep with the express intention that users should copy and paste this text into their manuscripts unchanged. It is released under the CC0 license.

**Supplementary Figure 1**. Temporal network measures at the trial time-scale (averaged over subjects and trials) for (**A**) SID, (**B**) within- and (**C**) between network degree centrality.

**

**Supplementary Figure 2**. Group-averaged temporal network measures at the trial time scale for each thermal intensity. (**A**) SID and (**B**) within- and between-network degree centrality. Each datapoint indicate group-averaged data.

**Supplementary Figure 3**. Group-averaged temporal network measures at the singe time-point scale for each thermal intensity. (**A**) SID, (**B**) within- and (**C**) between-network degree centrality.

**Supplementary Table 1**. Result of the correlation between SID values at the trial time-scale and subjective ratings of thermal stimuli. Degrees-of-freedom = 1100.

| Network | Outliers | *r* | 95% CI | *p*-uncorr | *p*-corr |
| --- | --- | --- | --- | --- | --- |
| Vis | 51 | -0.091 | [-0.15, -0.03] | 0.003 | 0.023 |
| SM | 63 | -0.058 | [-0.12, 0.00] | 0.060 | 0.104 |
| DA | 59 | -0.047 | [-0.11, 0.01] | 0.129 | 0.175 |
| SA | 63 | -0.078 | [-0.14, -0.02] | 0.011 | 0.040 |
| Limbic | 69 | -0.071 | [-0.13, -0.01] | 0.022 | 0.051 |
| FP | 63 | -0.045 | [-0.10, 0.01] | 0.150 | 0.175 |
| DMN | 64 | -0.030 | [-0.09, 0.03] | 0.336 | 0.336 |

**Supplementary Table 2**. Result of the correlation between within-network degree centrality at the trial time-scale and subjective ratings of thermal stimuli. Degrees-of-freedom = 1100.

| Network | Outliers | *r* | 95% CI | *p*-uncorr | *p*-corr |  |
| --- | --- | --- | --- | --- | --- | --- |
| Vis | | 72 | -0.079 | [-0.14, -0.02] | 0.011 | 0.077 |
| SM | | 63 | 0.024 | [-0.04, 0.08] | 0.449 | 0.762 |
| DA | | 58 | -0.014 | [-0.07, 0.05] | 0.649 | 0.762 |
| SA | | 64 | -0.006 | [-0.06, 0.05] | 0.859 | 0.859 |
| Limbic | | 51 | -0.056 | [-0.12, 0.00] | 0.068 | 0.214 |
| FP | | 52 | 0.052 | [-0.01, 0.11] | 0.092 | 0.214 |
| DMN | | 56 | 0.014 | [-0.05, 0.07] | 0.653 | 0.762 |

**Supplementary Table 3**. Result of the correlation between between-network degree centrality at the trial time-scale and subjective ratings of thermal stimuli. Degrees-of-freedom = 1100.

| Network | Outliers | *r* | 95% CI | *p*-uncorr | *p*-corr |
| --- | --- | --- | --- | --- | --- |
| Vis | 71 | -0.027 | [-0.09, 0.03] | 0.391 | 0.891 |
| SM | 71 | 0.015 | [-0.04, 0.07] | 0.630 | 0.891 |
| DA | 65 | 0.006 | [-0.05, 0.06] | 0.856 | 0.891 |
| SA | 73 | -0.007 | [-0.07, 0.05] | 0.811 | 0.891 |
| Limbic | 71 | -0.008 | [-0.07, 0.05] | 0.799 | 0.891 |
| FP | 68 | 0.037 | [-0.02, 0.10] | 0.239 | 0.891 |
| DMN | 58 | -0.004 | [-0.06, 0.05] | 0.891 | 0.891 |

**Supplementary Table 4**. Result of the skipped Spearman correlation (two-sided) correlation between SID values at single time-points and subjective ratings of thermal stimuli. Degrees-of-freedom = 1100.

| Network | Time | Outliers | *r* | 95% CI | *p*-uncorr | | *p*-corr |
| --- | --- | --- | --- | --- | --- | --- | --- |
|  | 1 | 0 | -0.016 | [-0.07, 0.04] | | 0.600 | 0.684 |
|  | 2 | 0 | -0.017 | [-0.08, 0.04] | | 0.566 | 0.660 |
|  | 3 | 0 | -0.046 | [-0.10, 0.01] | | 0.129 | 0.224 |
| Visual | 4 | 0 | -0.074 | [-0.13, -0.02] | | 0.014 | 0.039 |
|  | 5 | 15 | -0.083 | [-0.14, -0.02] | | 0.006 | 0.025 |
|  | 6 | 0 | -0.099 | [-0.16, -0.04] | | 0.001 | 0.012 |
|  | 7 | 0 | -0.083 | [-0.14, -0.02] | | 0.006 | 0.025 |
|  | 1 | 0 | 0.030 | [-0.03, 0.09] | | 0.325 | 0.419 |
|  | 2 | 0 | 0.018 | [-0.04, 0.08] | | 0.557 | 0.660 |
|  | 3 | 0 | -0.058 | [-0.12, 0.00] | | 0.054 | 0.123 |
| Somatomotor | 4 | 0 | -0.075 | [-0.13, -0.02] | | 0.012 | 0.039 |
|  | 5 | 0 | -0.109 | [-0.17, -0.05] | | 0.000 | 0.007 |
|  | 6 | 0 | -0.087 | [-0.15, -0.03] | | 0.004 | 0.025 |
|  | 7 | 0 | -0.067 | [-0.13, -0.01] | | 0.026 | 0.072 |
|  | 1 | 21 | 0.037 | [-0.02, 0.10] | | 0.225 | 0.315 |
|  | 2 | 0 | -0.009 | [-0.07, 0.05] | | 0.765 | 0.799 |
|  | 3 | 0 | -0.062 | [-0.12, -0.00] | | 0.039 | 0.096 |
| Dorsal-attention | 4 | 0 | -0.084 | [-0.14, -0.02] | | 0.006 | 0.025 |
|  | 5 | 0 | -0.088 | [-0.15, -0.03] | | 0.004 | 0.025 |
|  | 6 | 48 | -0.081 | [-0.14, -0.02] | | 0.009 | 0.034 |
|  | 7 | 0 | -0.075 | [-0.13, -0.02] | | 0.013 | 0.039 |
|  | 1 | 0 | 0.031 | [-0.03, 0.09] | | 0.303 | 0.402 |
|  | 2 | 0 | 0.025 | [-0.03, 0.08] | | 0.404 | 0.507 |
|  | 3 | 4 | -0.041 | [-0.10, 0.02] | | 0.173 | 0.257 |
| Salience | 4 | 0 | -0.053 | [-0.11, 0.01] | | 0.080 | 0.165 |
|  | 5 | 0 | -0.085 | [-0.14, -0.03] | | 0.005 | 0.025 |
|  | 6 | 0 | -0.044 | [-0.10, 0.01] | | 0.141 | 0.225 |
|  | 7 | 0 | -0.037 | [-0.10, 0.02] | | 0.224 | 0.315 |
|  | 1 | 0 | -0.013 | [-0.07, 0.05] | | 0.662 | 0.720 |
|  | 2 | 0 | -0.003 | [-0.06, 0.06] | | 0.918 | 0.937 |
|  | 3 | 0 | -0.046 | [-0.11, 0.01] | | 0.125 | 0.224 |
| Limbic | 4 | 0 | -0.065 | [-0.12, -0.01] | | 0.030 | 0.077 |
|  | 5 | 0 | -0.088 | [-0.15, -0.03] | | 0.004 | 0.025 |
|  | 6 | 0 | -0.044 | [-0.10, 0.01] | | 0.142 | 0.225 |
|  | 7 | 0 | -0.032 | [-0.09, 0.03] | | 0.296 | 0.402 |
|  | 1 | 0 | 0.023 | [-0.04, 0.08] | | 0.448 | 0.548 |
|  | 2 | 0 | 0.013 | [-0.05, 0.07] | | 0.657 | 0.720 |
|  | 3 | 0 | -0.053 | [-0.11, 0.01] | | 0.081 | 0.165 |
| Fronto-parietal | 4 | 0 | -0.085 | [-0.14, -0.03] | | 0.005 | 0.025 |
|  | 5 | 0 | -0.125 | [-0.18, -0.07] | | 0.000 | 0.002 |
|  | 6 | 0 | -0.077 | [-0.14, -0.02] | | 0.010 | 0.037 |
|  | 7 | 0 | -0.042 | [-0.10, 0.02] | | 0.164 | 0.252 |
|  | 1 | 0 | 0.001 | [-0.06, 0.06] | | 0.984 | 0.984 |
|  | 2 | 9 | 0.009 | [-0.05, 0.07] | | 0.766 | 0.799 |
|  | 3 | 0 | -0.058 | [-0.12, 0.00] | | 0.055 | 0.123 |
| Default-mode | 4 | 0 | -0.051 | [-0.11, 0.01] | | 0.094 | 0.183 |
|  | 5 | 32 | -0.102 | [-0.16, -0.04] | | 0.001 | 0.012 |
|  | 6 | 0 | -0.045 | [-0.10, 0.01] | | 0.133 | 0.224 |
|  | 7 | 0 | -0.047 | [-0.11, 0.01] | | 0.116 | 0.218 |

*Note*: p-values are corrected for multiple comparisons with False Discovery Rate.

**Supplementary Table 5**. Result of the skipped Spearman correlation (two-sided) between within-network degree centrality at single time-points and subjective ratings of thermal stimuli. Degrees-of-freedom = 1100.

| Network | Time | Outliers | *r* | 95% CI | *p*-uncorr | *p*-corr |
| --- | --- | --- | --- | --- | --- | --- |
|  | 1 | 84 | -0.052 | [-0.11, 0.01] | 0.096 | 0.293 |
|  | 2 | 76 | -0.033 | [-0.09, 0.03] | 0.287 | 0.557 |
|  | 3 | 89 | -0.032 | [-0.09, 0.03] | 0.307 | 0.557 |
| Visual | 4 | 84 | -0.030 | [-0.09, 0.03] | 0.341 | 0.596 |
|  | 5 | 88 | -0.011 | [-0.07, 0.05] | 0.737 | 0.802 |
|  | 6 | 91 | 0.033 | [-0.03, 0.09] | 0.300 | 0.557 |
|  | 7 | 88 | 0.037 | [-0.02, 0.10] | 0.236 | 0.557 |
|  | 1 | 86 | -0.012 | [-0.07, 0.05] | 0.697 | 0.776 |
|  | 2 | 73 | 0.035 | [-0.02, 0.09] | 0.260 | 0.557 |
|  | 3 | 73 | 0.020 | [-0.04, 0.08] | 0.525 | 0.671 |
| Somatomotor | 4 | 91 | 0.060 | [0.00, 0.12] | 0.055 | 0.275 |
|  | 5 | 86 | 0.036 | [-0.02, 0.09] | 0.257 | 0.557 |
|  | 6 | 84 | 0.001 | [-0.06, 0.06] | 0.977 | 0.990 |
|  | 7 | 81 | 0.019 | [-0.04, 0.08] | 0.548 | 0.671 |
|  | 1 | 89 | -0.080 | [-0.14,-0.02] | 0.011 | 0.135 |
|  | 2 | 67 | -0.047 | [-0.11, 0.01] | 0.129 | 0.373 |
|  | 3 | 78 | -0.024 | [-0.08, 0.03] | 0.438 | 0.663 |
| Dorsal-attention | 4 | 81 | 0.003 | [-0.06, 0.06] | 0.926 | 0.972 |
|  | 5 | 95 | 0.077 | [0.02, 0.14] | 0.015 | 0.145 |
|  | 6 | 67 | 0.056 | [-0.00, 0.11] | 0.071 | 0.275 |
|  | 7 | 73 | 0.020 | [-0.04, 0.08] | 0.517 | 0.671 |
|  | 1 | 86 | -0.066 | [-0.12,-0.01] | 0.037 | 0.268 |
|  | 2 | 85 | -0.038 | [-0.10, 0.02] | 0.226 | 0.557 |
|  | 3 | 69 | -0.013 | [-0.07, 0.05] | 0.670 | 0.771 |
| Salience | 4 | 80 | 0.023 | [-0.04, 0.08] | 0.460 | 0.663 |
|  | 5 | 80 | 0.027 | [-0.03, 0.09] | 0.387 | 0.654 |
|  | 6 | 101 | 0.022 | [-0.04, 0.08] | 0.491 | 0.671 |
|  | 7 | 81 | 0.035 | [-0.02, 0.09] | 0.265 | 0.557 |
|  | 1 | 75 | -0.021 | [-0.08, 0.04] | 0.499 | 0.671 |
|  | 2 | 72 | -0.052 | [-0.11, 0.01] | 0.095 | 0.293 |
|  | 3 | 82 | -0.103 | [-0.16,-0.04] | 0.001 | 0.049 |
| Limbic | 4 | 94 | -0.091 | [-0.15,-0.03] | 0.004 | 0.065 |
|  | 5 | 79 | -0.057 | [-0.12, 0.00] | 0.069 | 0.275 |
|  | 6 | 77 | -0.093 | [-0.15,-0.03] | 0.003 | 0.065 |
|  | 7 | 86 | -0.065 | [-0.12,-0.01] | 0.038 | 0.268 |
|  | 1 | 74 | 0.055 | [-0.00, 0.11] | 0.078 | 0.275 |
|  | 2 | 73 | 0.024 | [-0.04, 0.08] | 0.440 | 0.663 |
|  | 3 | 82 | 0.003 | [-0.06, 0.06] | 0.933 | 0.972 |
| Fronto-parietal | 4 | 76 | -0.013 | [-0.07, 0.05] | 0.676 | 0.771 |
|  | 5 | 75 | 0.056 | [-0.00, 0.11] | 0.074 | 0.275 |
|  | 6 | 76 | 0.063 | [0.00, 0.12] | 0.044 | 0.269 |
|  | 7 | 79 | 0.033 | [-0.03, 0.09] | 0.299 | 0.557 |
|  | 1 | 82 | 0.019 | [-0.04, 0.08] | 0.542 | 0.671 |
|  | 2 | 75 | 0.025 | [-0.03, 0.08] | 0.431 | 0.663 |
|  | 3 | 72 | -0.057 | [-0.12, 0.00] | 0.066 | 0.275 |
| Default-mode | 4 | 68 | -0.013 | [-0.07, 0.05] | 0.668 | 0.771 |
|  | 5 | 76 | 0.046 | [-0.01, 0.11] | 0.137 | 0.373 |
|  | 6 | 74 | 0.000 | [-0.06, 0.06] | 0.990 | 0.990 |
|  | 7 | 80 | -0.023 | [-0.08, 0.04] | 0.455 | 0.663 |

*Note*: p-values are corrected for multiple comparisons with False Discovery Rate.

**Supplementary Table 6** Result of the skipped Spearman correlation (two-sided) correlation between between-network degree centrality at single time-points and subjective ratings of thermal stimuli. Degrees-of-freedom = 1100.

| Network | Time | Outliers | *r* | 95% CI | *p*-uncorr | *p*-corr |
| --- | --- | --- | --- | --- | --- | --- |
|  | 1 | 81 | -0.059 | [-0.12, 0.00] | 0.060 | 0.173 |
|  | 2 | 73 | -0.036 | [-0.09, 0.02] | 0.249 | 0.508 |
|  | 3 | 86 | -0.016 | [-0.08, 0.04] | 0.609 | 0.746 |
| Visual | 4 | 86 | -0.029 | [-0.09, 0.03] | 0.354 | 0.562 |
|  | 5 | 86 | 0.044 | [-0.01, 0.10] | 0.159 | 0.375 |
|  | 6 | 86 | 0.122 | [0.06, 0.18] | 0.000 | 0.005 |
|  | 7 | 81 | 0.072 | [0.01, 0.13] | 0.022 | 0.106 |
|  | 1 | 89 | -0.035 | [-0.09, 0.02] | 0.272 | 0.511 |
|  | 2 | 78 | 0.020 | [-0.04, 0.08] | 0.529 | 0.675 |
|  | 3 | 80 | 0.033 | [-0.03, 0.09] | 0.296 | 0.518 |
| Somatomotor | 4 | 88 | 0.034 | [-0.03, 0.09] | 0.281 | 0.511 |
|  | 5 | 90 | 0.080 | [0.02, 0.14] | 0.011 | 0.060 |
|  | 6 | 85 | 0.064 | [0.00, 0.12] | 0.042 | 0.158 |
|  | 7 | 89 | 0.081 | [0.02, 0.14] | 0.010 | 0.060 |
|  | 1 | 78 | -0.053 | [-0.11, 0.01] | 0.089 | 0.241 |
|  | 2 | 77 | -0.007 | [-0.07, 0.05] | 0.813 | 0.945 |
|  | 3 | 88 | 0.028 | [-0.03, 0.09] | 0.367 | 0.562 |
| Dorsal-attention | 4 | 91 | 0.029 | [-0.03, 0.09] | 0.360 | 0.562 |
|  | 5 | 94 | 0.104 | [0.05, 0.16] | 0.001 | 0.012 |
|  | 6 | 88 | 0.108 | [0.05, 0.17] | 0.001 | 0.009 |
|  | 7 | 83 | 0.083 | [0.02, 0.14] | 0.008 | 0.055 |
|  | 1 | 86 | -0.044 | [-0.10, 0.02] | 0.161 | 0.375 |
|  | 2 | 85 | -0.021 | [-0.08, 0.04] | 0.503 | 0.675 |
|  | 3 | 75 | 0.003 | [-0.06, 0.06] | 0.926 | 0.975 |
| Salience | 4 | 90 | -0.002 | [-0.06, 0.06] | 0.953 | 0.975 |
|  | 5 | 85 | 0.039 | [-0.02, 0.10] | 0.213 | 0.454 |
|  | 6 | 88 | 0.060 | [0.00, 0.12] | 0.055 | 0.173 |
|  | 7 | 85 | 0.085 | [0.03, 0.14] | 0.007 | 0.055 |
|  | 1 | 84 | 0.030 | [-0.03, 0.09] | 0.345 | 0.562 |
|  | 2 | 77 | -0.020 | [-0.08, 0.04] | 0.529 | 0.675 |
|  | 3 | 83 | 0.007 | [-0.05, 0.07] | 0.829 | 0.945 |
| Limbic | 4 | 79 | 0.003 | [-0.06, 0.06] | 0.931 | 0.975 |
|  | 5 | 68 | 0.050 | [-0.01, 0.11] | 0.108 | 0.278 |
|  | 6 | 78 | 0.069 | [0.01, 0.13] | 0.027 | 0.112 |
|  | 7 | 81 | 0.020 | [-0.04, 0.08] | 0.530 | 0.675 |
|  | 1 | 89 | 0.003 | [-0.06, 0.06] | 0.923 | 0.975 |
|  | 2 | 83 | 0.008 | [-0.05, 0.07] | 0.794 | 0.945 |
|  | 3 | 86 | 0.002 | [-0.06, 0.06] | 0.955 | 0.975 |
| Fronto-parietal | 4 | 79 | 0.034 | [-0.03, 0.09] | 0.278 | 0.511 |
|  | 5 | 81 | 0.095 | [0.04, 0.15] | 0.002 | 0.023 |
|  | 6 | 92 | 0.113 | [0.05, 0.17] | 0.000 | 0.008 |
|  | 7 | 94 | 0.060 | [0.00, 0.12] | 0.057 | 0.173 |
|  | 1 | 79 | -0.026 | [-0.08, 0.03] | 0.407 | 0.604 |
|  | 2 | 81 | -0.019 | [-0.08, 0.04] | 0.537 | 0.675 |
|  | 3 | 80 | -0.001 | [-0.06, 0.06] | 0.975 | 0.975 |
| Default-mode | 4 | 72 | -0.025 | [-0.08, 0.03] | 0.422 | 0.608 |
|  | 5 | 74 | 0.061 | [0.00, 0.12] | 0.053 | 0.173 |
|  | 6 | 81 | 0.069 | [0.01, 0.13] | 0.027 | 0.112 |
|  | 7 | 84 | 0.042 | [-0.02, 0.10] | 0.182 | 0.406 |

*Note*: p-values are corrected for multiple comparisons with False Discovery Rate.

*Analysis without global signal regression*

In time-varying functional connectivity analysis, preprocessing methods that include global signal regression has been shown to be the most effective de-noising strategies (Lydon-Staley et al. 2019). However, the global signal regression is known to induce spurious negative correlations (ref). For the sake of completeness, we re-analysed all results without global signal regression.

**Supplementary Figure 4**. Estimates of SID (A), within- (B) and between-network degree centrality (C) averaged over all trials. The default-mode network was the only network that showed statistically significant difference in SID between high and low thermal intensities but was not significant after correcting for multiple comparisons (estimate: -0.128, *p* = 0.04, *p*_corr_ = 0.17). See Table 7 for details for all networks for the SID measure. There no statistically significant results for within- and between-network degree centrality (Tables 8,9). When we had regressed the global signal, the results suggested that the fronto-parietal network integrated more for higher thermal intensities when aggregated over trials (uncorrected). Here, the result suggests that the default-mode network integrates more (uncorrected). The uncorrected p-value for the fronto-parietal network is *p* = 0.059.

**Supplementary Table 7**. Results for Segregation Integration Difference (SID). A permutation test was implemented to compare response to high and low thermal intensities.

| Network | *Est* | *p*-uncorr | *p*-corr |
| --- | --- | --- | --- |
| Vis | -0.073 | 0.139 | 0.195 |
| SM | -0.070 | 0.242 | 0.283 |
| DA | -0.084 | 0.077 | 0.178 |
| SA | -0.093 | 0.101 | 0.178 |
| Limbic | -0.008 | 0.916 | 0.916 |
| FP | -0.123 | 0.059 | 0.178 |
| DMN | -0.128 | 0.040 | 0.178 |

**Supplementary Table 8**. Results for the within-network degree centrality. A permutation test was implemented to compare response to high and low thermal intensities.

| Network | *Est* | *p*-uncorr | *p*-corr |
| --- | --- | --- | --- |
| Vis | -38.89 | 0.593 | NA |
| SM | 2.82 | 0.973 | NA |
| DA | 9.05 | 0.827 | NA |
| SA | 0.44 | 0.992 | NA |
| Limbic | -5.56 | 0.354 | NA |
| FP | 14.43 | 0.736 | NA |
| DMN | -72.98 | 0.539 | NA |

**Supplementary Table 9**. Results for the between-network degree centrality. A permutation test was implemented to compare response to high and low thermal intensities.

| Network | *Est* | *p*-uncorr | *p*-corr |
| --- | --- | --- | --- |
| Vis | 1.54 | 0.973 | NA |
| SM | 22.04 | 0.715 | NA |
| DA | 19.68 | 0.644 | NA |
| SA | 17.34 | 0.654 | NA |
| Limbic | -6.81 | 0.681 | NA |
| FP | 29.05 | 0.481 | NA |
| DMN | 26.71 | 0.648 | NA |

**
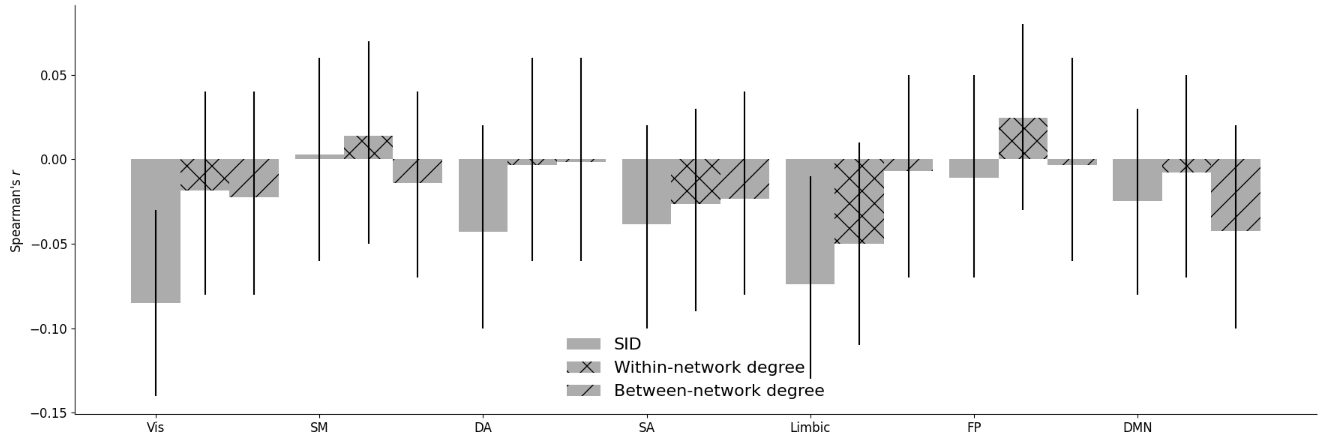
**

**Supplementary Figure 5**. The strength of the statistical relation between temporal network measures (SID, within- and between-network degree centrality) and pain ratings. The degree of correlation between pain ratings and each temporal network measure was statistically significant for the visual (*r* = -0.085, *p_un_*_corr_ = 0.006, *p*_corr_ = 0.041). See Supplementary Tables 10-12 for detailed results for each network.

**Supplementary Table 10**. Result of the correlation between SID values at the trial time-scale and subjective ratings of thermal stimuli. Degrees-of-freedom = 1100.

| Network | Outliers | *r* | 95% CI | *p*-uncorr | *p*-corr |
| --- | --- | --- | --- | --- | --- |
| Vis | 57 | -0.085 | [-0.14, -0.03] | 0.006 | 0.041 |
| SM | 68 | 0.003 | [-0.06, 0.06] | 0.932 | 0.932 |
| DA | 49 | -0.043 | [-0.10, 0.02] | 0.163 | 0.373 |
| SA | 58 | -0.039 | [-0.10, 0.02] | 0.213 | 0.373 |
| Limbic | 68 | -0.074 | [-0.13, -0.01] | 0.018 | 0.062 |
| FP | 60 | -0.011 | [-0.07, 0.05] | 0.730 | 0.852 |
| DMN | 65 | -0.025 | [-0.08, 0.03] | 0.426 | 0.597 |

**Supplementary Table 11**. Result of the correlation between within-network degree centrality at the trial time-scale and subjective ratings of thermal stimuli. Degrees-of-freedom = 1100.

| Network | Outliers | *r* | 95% CI | *p*-uncorr | *p*-corr |  |
| --- | --- | --- | --- | --- | --- | --- |
| Vis | | 65 | -0.018 | [-0.08, 0.04] | 0.553 | 0.912 |
| SM | | 65 | 0.014 | [-0.05, 0.07] | 0.656 | 0.912 |
| DA | | 59 | -0.003 | [-0.06, 0.06] | 0.912 | 0.912 |
| SA | | 69 | -0.026 | [-0.09, 0.03] | 0.397 | 0.912 |
| Limbic | | 66 | -0.050 | [-0.11, 0.01] | 0.109 | 0.760 |
| FP | | 62 | 0.024 | [-0.03, 0.08] | 0.431 | 0.912 |
| DMN | | 59 | -0.008 | [-0.07, 0.05] | 0.802 | 0.912 |

**Supplementary Table 12**. Result of the correlation between between-network degree centrality at the trial time-scale and subjective ratings of thermal stimuli. Degrees-of-freedom = 1100.

| Network | Outliers | *r* | 95% CI | *p*-uncorr | *p*-corr |
| --- | --- | --- | --- | --- | --- |
| Vis | 62 | -0.023 | [-0.08, 0.04] | 0.469 | 0.961 |
| SM | 66 | -0.014 | [-0.07, 0.04] | 0.649 | 0.961 |
| DA | 63 | -0.002 | [-0.06, 0.06] | 0.961 | 0.961 |
| SA | 63 | -0.023 | [-0.08, 0.04] | 0.450 | 0.961 |
| Limbic | 70 | -0.007 | [-0.07, 0.05] | 0.828 | 0.961 |
| FP | 69 | -0.003 | [-0.06, 0.06] | 0.918 | 0.961 |
| DMN | 65 | -0.042 | [-0.10, 0.02] | 0.173 | 0.961 |

**Supplementary Figure 6**. This figure shows SID estimates (**A**), within- (**B**) and between-network degree centrality (**C**). Shown in A-C is the average across participants and trials for the seven volumes following thermal stimulation for the two different stimuli (“high” and “low”). Error bars show the 95% confidence interval. There was a statistically significant difference in SID between high and low thermal intensities for the visual (Vis), salience network (SA) and default-mode network (DMN). Only the default-mode network showed statistically significant difference at one time-point that survived multiple comparisons (Vis: time-point = 6, *F* = 1.72, *p* = 0.024, *p*_corr_ = 0.100; SA: time-point = 6, *F* = 1.68, *p* = 0.029, *p*_corr_ = 0.100; DMN: time-point = 6, *F* = 2.40, *p* = 0.007, *p*_corr_ = 0.036). There were no statistically significant results for within- and between-network degree centrality.

**Supplementary Figure 7**. The relationship between temporal network parameters at different time-points during trials of thermal stimulation (**A** – SID; **B** – within-network degree centrality; **C** – between-network degree centrality) and subjective ratings of the stimuli. Error bars show the 95% confidence interval. Red bars represent time-points with statistically significant correlation (*p* < 0.05, corrected across time points and brain networks). There is more integration with higher pain ratings for the visual, dorsal attention, salience, frontoparietal and default-mode networks (*p*_corr_ < 0.05). There was no statistical association for the within- network degree centrality. Only the visual network showed statistically significant correlation to pain ratings for the between-network degree centrality (*p*_corr_ < 0.05). Supplementary Tables 13-15 shows detailed results for each measure.

**Supplementary Table 13**. Result of the correlation between SID values at single time-points and subjective ratings of thermal stimuli. Degrees-of-freedom = 1100.

| Network | Time | Outliers | *r* | 95% CI | *p*-uncorr | | *p*-corr |
| --- | --- | --- | --- | --- | --- | --- | --- |
|  | 1 | 0 | -0.050 | [-0.11, 0.01] | | 0.095 | 0.233 |
|  | 2 | 0 | -0.030 | [-0.09, 0.03] | | 0.326 | 0.484 |
|  | 3 | 0 | -0.053 | [-0.11, 0.01] | | 0.080 | 0.206 |
| Visual | 4 | 0 | -0.040 | [-0.10, 0.02] | | 0.186 | 0.326 |
|  | 5 | 0 | -0.057 | [-0.12, 0.00] | | 0.061 | 0.165 |
|  | 6 | 8 | -0.119 | [-0.18,-0.06] | | 0.000 | 0.002 |
|  | 7 | 1 | -0.087 | [-0.15, 0.03] | | 0.004 | 0.031 |
|  | 1 | 0 | 0.006 | [-0.05, 0.07] | | 0.842 | 0.938 |
|  | 2 | 0 | 0.000 | [-0.06, 0.06] | | 0.995 | 0.995 |
|  | 3 | 0 | -0.049 | [-0.11, 0.01] | | 0.107 | 0.250 |
| Somatomotor | 4 | 0 | -0.058 | [-0.12, 0.00] | | 0.053 | 0.164 |
|  | 5 | 0 | -0.063 | [-0.12,-0.00] | | 0.037 | 0.137 |
|  | 6 | 0 | -0.078 | [-0.14,-0.02] | | 0.010 | 0.060 |
|  | 7 | 0 | -0.078 | [-0.14,-0.02] | | 0.010 | 0.060 |
|  | 1 | 0 | -0.025 | [-0.08, 0.03] | | 0.412 | 0.594 |
|  | 2 | 4 | -0.007 | [-0.07, 0.05] | | 0.810 | 0.923 |
|  | 3 | 0 | -0.021 | [-0.08, 0.04] | | 0.488 | 0.664 |
| Dorsal-attention | 4 | 0 | -0.031 | [-0.09, 0.03] | | 0.306 | 0.469 |
|  | 5 | 0 | -0.042 | [-0.10, 0.02] | | 0.163 | 0.315 |
|  | 6 | 0 | -0.094 | [-0.15,-0.03] | | 0.002 | 0.022 |
|  | 7 | 0 | -0.062 | [-0.12,-0.00] | | 0.038 | 0.137 |
|  | 1 | 0 | 0.011 | [-0.05, 0.07] | | 0.715 | 0.855 |
|  | 2 | 0 | 0.000 | [-0.06, 0.06] | | 0.992 | 0.995 |
|  | 3 | 19 | -0.044 | [-0.10, 0.01] | | 0.147 | 0.313 |
| Salience | 4 | 0 | -0.018 | [-0.08, 0.04] | | 0.543 | 0.700 |
|  | 5 | 0 | -0.074 | [-0.13,-0.01] | | 0.014 | 0.078 |
|  | 6 | 0 | -0.126 | [-0.18,-0.07] | | 0.000 | 0.001 |
|  | 7 | 0 | -0.035 | [-0.09, 0.02] | | 0.248 | 0.420 |
|  | 1 | 0 | -0.041 | [-0.10, 0.02] | | 0.173 | 0.315 |
|  | 2 | 0 | 0.000 | [-0.06, 0.06] | | 0.991 | 0.995 |
|  | 3 | 0 | -0.041 | [-0.10, 0.02] | | 0.169 | 0.315 |
| Limbic | 4 | 0 | -0.033 | [-0.09, 0.03] | | 0.277 | 0.446 |
|  | 5 | 0 | -0.032 | [-0.09, 0.03] | | 0.282 | 0.446 |
|  | 6 | 0 | -0.061 | [-0.12,-0.00] | | 0.042 | 0.137 |
|  | 7 | 2 | -0.062 | [-0.12,-0.00] | | 0.040 | 0.137 |
|  | 1 | 0 | -0.015 | [-0.07, 0.04] | | 0.617 | 0.775 |
|  | 2 | 0 | 0.010 | [-0.05, 0.07] | | 0.744 | 0.868 |
|  | 3 | 0 | -0.019 | [-0.08, 0.04] | | 0.529 | 0.700 |
| Fronto-parietal | 4 | 0 | -0.041 | [-0.10, 0.02] | | 0.173 | 0.315 |
|  | 5 | 0 | -0.069 | [-0.13,-0.01] | | 0.021 | 0.104 |
|  | 6 | 0 | -0.101 | [-0.16,-0.04] | | 0.001 | 0.014 |
|  | 7 | 0 | -0.068 | [-0.13,-0.01] | | 0.024 | 0.105 |
|  | 1 | 0 | -0.005 | [-0.06, 0.05] | | 0.881 | 0.960 |
|  | 2 | 0 | -0.012 | [-0.07, 0.05] | | 0.691 | 0.846 |
|  | 3 | 0 | -0.024 | [-0.08, 0.04] | | 0.432 | 0.604 |
| Default-mode | 4 | 18 | 0.000 | [-0.06, 0.06] | | 0.987 | 0.995 |
|  | 5 | 0 | -0.045 | [-0.10, 0.01] | | 0.137 | 0.306 |
|  | 6 | 0 | -0.088 | [-0.15,-0.03] | | 0.004 | 0.031 |
|  | 7 | 0 | -0.057 | [-0.12, 0.00] | | 0.059 | 0.165 |

*Note*: p-values are corrected for multiple comparisons with False Discovery Rate.

**Supplementary Table 14**. Result of the correlation between within-network degree centrality at single time-points and subjective ratings of thermal stimuli. Degrees-of-freedom = 1100.

| Network | Time | Outliers | *r* | 95% CI | *p*-uncorr | *p*-corr |
| --- | --- | --- | --- | --- | --- | --- |
|  | 1 | 69 | -0.037 | [-0.10, 0.02] | 0.236 | 0.652 |
|  | 2 | 80 | -0.015 | [-0.07, 0.04] | 0.636 | 0.843 |
|  | 3 | 78 | -0.033 | [-0.09, 0.03] | 0.298 | 0.652 |
| Visual | 4 | 84 | 0.008 | [-0.05, 0.07] | 0.800 | 0.907 |
|  | 5 | 85 | 0.028 | [-0.03, 0.09] | 0.370 | 0.658 |
|  | 6 | 94 | 0.074 | [0.02, 0.13] | 0.018 | 0.205 |
|  | 7 | 77 | 0.031 | [-0.03, 0.09] | 0.329 | 0.658 |
|  | 1 | 78 | 0.022 | [-0.04, 0.08] | 0.492 | 0.715 |
|  | 2 | 88 | 0.010 | [-0.05, 0.07] | 0.751 | 0.907 |
|  | 3 | 90 | -0.010 | [-0.07, 0.05] | 0.760 | 0.907 |
| Somatomotor | 4 | 99 | 0.007 | [-0.05, 0.07] | 0.823 | 0.907 |
|  | 5 | 94 | 0.044 | [-0.01, 0.10] | 0.160 | 0.652 |
|  | 6 | 90 | 0.044 | [-0.01, 0.10] | 0.160 | 0.652 |
|  | 7 | 78 | -0.019 | [-0.08, 0.04] | 0.540 | 0.735 |
|  | 1 | 71 | -0.038 | [-0.10, 0.02] | 0.228 | 0.652 |
|  | 2 | 85 | -0.005 | [-0.06, 0.05] | 0.880 | 0.907 |
|  | 3 | 79 | -0.002 | [-0.06, 0.06] | 0.938 | 0.938 |
| Dorsal-attention | 4 | 76 | 0.005 | [-0.05, 0.06] | 0.875 | 0.907 |
|  | 5 | 91 | 0.032 | [-0.03, 0.09] | 0.306 | 0.652 |
|  | 6 | 91 | 0.042 | [-0.02, 0.10] | 0.184 | 0.652 |
|  | 7 | 94 | -0.024 | [-0.08, 0.04] | 0.453 | 0.707 |
|  | 1 | 81 | -0.038 | [-0.10, 0.02] | 0.222 | 0.652 |
|  | 2 | 73 | -0.021 | [-0.08, 0.04] | 0.500 | 0.715 |
|  | 3 | 71 | -0.033 | [-0.09, 0.03] | 0.292 | 0.652 |
| Salience | 4 | 90 | -0.028 | [-0.09, 0.03] | 0.371 | 0.658 |
|  | 5 | 104 | -0.028 | [-0.09, 0.03] | 0.385 | 0.658 |
|  | 6 | 97 | -0.014 | [-0.07, 0.05] | 0.665 | 0.857 |
|  | 7 | 80 | 0.034 | [-0.03, 0.09] | 0.276 | 0.652 |
|  | 1 | 75 | 0.007 | [-0.05, 0.07] | 0.812 | 0.907 |
|  | 2 | 82 | -0.011 | [-0.07, 0.05] | 0.728 | 0.907 |
|  | 3 | 81 | -0.056 | [-0.11, 0.00] | 0.074 | 0.607 |
| Limbic | 4 | 86 | -0.052 | [-0.11, 0.01] | 0.096 | 0.652 |
|  | 5 | 88 | -0.038 | [-0.10, 0.02] | 0.226 | 0.652 |
|  | 6 | 86 | -0.027 | [-0.09, 0.03] | 0.389 | 0.658 |
|  | 7 | 91 | -0.091 | [-0.15,-0.03] | 0.004 | 0.060 |
|  | 1 | 81 | 0.042 | [-0.02, 0.10] | 0.183 | 0.652 |
|  | 2 | 80 | 0.095 | [0.04, 0.15] | 0.002 | 0.060 |
|  | 3 | 84 | 0.023 | [-0.04, 0.08] | 0.461 | 0.707 |
| Fronto-parietal | 4 | 90 | -0.048 | [-0.11, 0.01] | 0.130 | 0.652 |
|  | 5 | 71 | 0.035 | [-0.02, 0.09] | 0.265 | 0.652 |
|  | 6 | 71 | 0.072 | [0.01, 0.13] | 0.021 | 0.205 |
|  | 7 | 73 | -0.021 | [-0.08, 0.04] | 0.510 | 0.715 |
|  | 1 | 71 | -0.030 | [-0.09, 0.03] | 0.337 | 0.658 |
|  | 2 | 70 | -0.035 | [-0.09, 0.02] | 0.260 | 0.652 |
|  | 3 | 84 | -0.040 | [-0.10, 0.02] | 0.204 | 0.652 |
| Default-mode | 4 | 89 | -0.024 | [-0.08, 0.03] | 0.439 | 0.707 |
|  | 5 | 79 | 0.091 | [0.03, 0.15] | 0.004 | 0.060 |
|  | 6 | 78 | 0.004 | [-0.05, 0.06] | 0.888 | 0.907 |
|  | 7 | 85 | 0.005 | [-0.05, 0.06] | 0.872 | 0.907 |

*Note*: p-values are corrected for multiple comparisons with False Discovery Rate.

**Supplementary Table 15** Result of the correlation between between-network degree centrality at single time-points and subjective ratings of thermal stimuli. Degrees-of-freedom = 1100.

| Network | Time | Outliers | *r* | 95% CI | *p*-uncorr | *p*-corr |
| --- | --- | --- | --- | --- | --- | --- |
|  | 1 | 83 | 0.011 | [-0.05, 0.07] | 0.724 | 0.953 |
|  | 2 | 83 | 0.004 | [-0.06, 0.06] | 0.901 | 0.981 |
|  | 3 | 89 | -0.047 | [-0.11, 0.01] | 0.138 | 0.465 |
| Visual | 4 | 80 | 0.005 | [-0.05, 0.06] | 0.880 | 0.980 |
|  | 5 | 101 | 0.028 | [-0.03, 0.09] | 0.382 | 0.703 |
|  | 6 | 99 | 0.108 | [0.05, 0.17] | 0.001 | 0.030 |
|  | 7 | 85 | 0.079 | [0.02, 0.14] | 0.012 | 0.188 |
|  | 1 | 76 | 0.045 | [-0.01, 0.10] | 0.154 | 0.471 |
|  | 2 | 82 | -0.006 | [-0.06, 0.05] | 0.856 | 0.980 |
|  | 3 | 90 | -0.035 | [-0.09, 0.02] | 0.263 | 0.679 |
| Somatomotor | 4 | 94 | 0.005 | [-0.05, 0.06] | 0.870 | 0.980 |
|  | 5 | 104 | 0.027 | [-0.03, 0.09] | 0.396 | 0.703 |
|  | 6 | 90 | 0.058 | [-0.00, 0.12] | 0.066 | 0.353 |
|  | 7 | 97 | 0.031 | [-0.03, 0.09] | 0.326 | 0.703 |
|  | 1 | 73 | 0.028 | [-0.03, 0.09] | 0.369 | 0.703 |
|  | 2 | 80 | 0.006 | [-0.05, 0.06] | 0.853 | 0.980 |
|  | 3 | 87 | -0.013 | [-0.07, 0.05] | 0.674 | 0.953 |
| Dorsal-attention | 4 | 84 | 0.000 | [-0.06, 0.06] | 0.991 | 0.991 |
|  | 5 | 103 | 0.011 | [-0.05, 0.07] | 0.739 | 0.953 |
|  | 6 | 108 | 0.084 | [0.02, 0.14] | 0.008 | 0.188 |
|  | 7 | 109 | 0.041 | [-0.02, 0.10] | 0.192 | 0.554 |
|  | 1 | 72 | -0.005 | [-0.06, 0.05] | 0.862 | 0.980 |
|  | 2 | 82 | 0.012 | [-0.05, 0.07] | 0.709 | 0.953 |
|  | 3 | 75 | -0.025 | [-0.08, 0.03] | 0.430 | 0.703 |
| Salience | 4 | 88 | -0.061 | [-0.12 -0.00] | 0.054 | 0.353 |
|  | 5 | 108 | -0.026 | [-0.08, 0.03] | 0.420 | 0.703 |
|  | 6 | 101 | 0.054 | [-0.01, 0.11] | 0.090 | 0.369 |
|  | 7 | 86 | 0.029 | [-0.03, 0.09] | 0.350 | 0.703 |
|  | 1 | 93 | 0.009 | [-0.05, 0.07] | 0.769 | 0.966 |
|  | 2 | 76 | -0.001 | [-0.06, 0.06] | 0.977 | 0.991 |
|  | 3 | 76 | -0.046 | [-0.10, 0.01] | 0.142 | 0.465 |
| Limbic | 4 | 95 | -0.057 | [-0.12, 0.00] | 0.070 | 0.353 |
|  | 5 | 94 | 0.002 | [-0.06, 0.06] | 0.939 | 0.991 |
|  | 6 | 80 | 0.059 | [-0.00, 0.12] | 0.061 | 0.353 |
|  | 7 | 79 | -0.027 | [-0.09, 0.03] | 0.388 | 0.703 |
|  | 1 | 79 | 0.075 | [0.02, 0.13] | 0.017 | 0.202 |
|  | 2 | 85 | 0.025 | [-0.03, 0.08] | 0.428 | 0.703 |
|  | 3 | 85 | -0.029 | [-0.09, 0.03] | 0.359 | 0.703 |
| Fronto-parietal | 4 | 91 | 0.011 | [-0.05, 0.07] | 0.736 | 0.953 |
|  | 5 | 82 | 0.051 | [-0.01, 0.11] | 0.104 | 0.394 |
|  | 6 | 83 | 0.055 | [-0.00, 0.11] | 0.077 | 0.353 |
|  | 7 | 88 | 0.013 | [-0.05, 0.07] | 0.680 | 0.953 |
|  | 1 | 77 | 0.027 | [-0.03, 0.09] | 0.397 | 0.703 |
|  | 2 | 81 | -0.002 | [-0.06, 0.06] | 0.954 | 0.991 |
|  | 3 | 81 | -0.055 | [-0.11, 0.00] | 0.079 | 0.353 |
| Default-mode | 4 | 80 | -0.038 | [-0.10, 0.02] | 0.227 | 0.618 |
|  | 5 | 84 | 0.022 | [-0.04, 0.08] | 0.486 | 0.768 |
|  | 6 | 85 | 0.073 | [0.01, 0.13] | 0.021 | 0.202 |
|  | 7 | 83 | 0.017 | [-0.04, 0.08] | 0.599 | 0.917 |

*Note*: p-values are corrected for multiple comparisons with False Discovery Rate.

**Supplementary Figure 8**. Comparison between temporal network measures after global signal regression (y-axis) and without global signal regression (x-axis). The left column shows data at the trial-time scale for (**A**) SID, (**B**) within-network degree centrality and (**C**) between-network degree centrality. One datapoint is the network property for one participant at one trial. The right column shows the data at the single time-point scale for (**D**) SID, (**E**) within-network degree centrality and (**F**) between-network degree centrality. One datapoint correspond to the network property for one participant and one time-point (one volume within a trial). This shows that the choice of global signal regression has an influence on positive values of each network metric, where the spread is larger. Focusing on the right column, panel D, the limbic network (purple) shows largest extreme values. In panels E and F, the somatomotor (orange) and default-mode network (magenta) shows the largest dispersion. This suggests that global signal regression mostly influences SID for the limbic network and the within- and between-network degree centrality for the somatomotor and default-mode network.

*fMRIPrep References*

Abraham, Alexandre, Fabian Pedregosa, Michael Eickenberg, Philippe Gervais, Andreas Mueller, Jean Kossaifi, Alexandre Gramfort, Bertrand Thirion, and Gael Varoquaux. 2014. “Machine Learning for Neuroimaging with Scikit-Learn.” Frontiers in Neuroinformatics 8. https://doi.org/10.3389/fninf.2014.00014.

Avants, B.B., C.L. Epstein, M. Grossman, and J.C. Gee. 2008. “Symmetric Diffeomorphic Image Registration with Cross-Correlation: Evaluating Automated Labeling of Elderly and Neurodegenerative Brain.” Medical Image Analysis 12 (1): 26–41. https://doi.org/10.1016/j.media.2007.06.004.

Behzadi, Yashar, Khaled Restom, Joy Liau, and Thomas T. Liu. 2007. “A Component Based Noise Correction Method (CompCor) for BOLD and Perfusion Based fMRI.” NeuroImage 37 (1): 90–101. https://doi.org/10.1016/j.neuroimage.2007.04.042.

Esteban, Oscar, Ross Blair, Christopher J. Markiewicz, Shoshana L. Berleant, Craig Moodie, Feilong Ma, Ayse Ilkay Isik, et al. 2018. “FMRIPrep.” Software. Zenodo. https://doi.org/10.5281/zenodo.852659.

Esteban, Oscar, Christopher Markiewicz, Ross W Blair, Craig Moodie, Ayse Ilkay Isik, Asier Erramuzpe Aliaga, James Kent, et al. 2018. “fMRIPrep: A Robust Preprocessing Pipeline for Functional MRI.” Nature Methods. https://doi.org/10.1038/s41592-018-0235-4.

Fonov, VS, AC Evans, RC McKinstry, CR Almli, and DL Collins. 2009. “Unbiased Nonlinear Average Age-Appropriate Brain Templates from Birth to Adulthood.” NeuroImage 47, Supplement 1: S102. https://doi.org/10.1016/S1053-8119(09)70884-5.

Gorgolewski, K., C. D. Burns, C. Madison, D. Clark, Y. O. Halchenko, M. L. Waskom, and S. Ghosh. 2011. “Nipype: A Flexible, Lightweight and Extensible Neuroimaging Data Processing Framework in Python.” Frontiers in Neuroinformatics 5: 13. https://doi.org/10.3389/fninf.2011.00013.

Gorgolewski, Krzysztof J., Oscar Esteban, Christopher J. Markiewicz, Erik Ziegler, David Gage Ellis, Michael Philipp Notter, Dorota Jarecka, et al. 2018. “Nipype.” Software. Zenodo. https://doi.org/10.5281/zenodo.596855.

Greve, Douglas N, and Bruce Fischl. 2009. “Accurate and Robust Brain Image Alignment Using Boundary-Based Registration.” NeuroImage 48 (1): 63–72. https://doi.org/10.1016/j.neuroimage.2009.06.060.

Jenkinson, Mark, Peter Bannister, Michael Brady, and Stephen Smith. 2002. “Improved Optimization for the Robust and Accurate Linear Registration and Motion Correction of Brain Images.” NeuroImage 17 (2): 825–41. https://doi.org/10.1006/nimg.2002.1132.

Jenkinson, Mark, and Stephen Smith. 2001. “A Global Optimisation Method for Robust Affine Registration of Brain Images.” Medical Image Analysis 5 (2): 143–56. https://doi.org/10.1016/S1361-8415(01)00036-6.

Lanczos, C. 1964. “Evaluation of Noisy Data.” Journal of the Society for Industrial and Applied Mathematics Series B Numerical Analysis 1 (1): 76–85. https://doi.org/10.1137/0701007.

Power, Jonathan D., Anish Mitra, Timothy O. Laumann, Abraham Z. Snyder, Bradley L. Schlaggar, and Steven E. Petersen. 2014. “Methods to Detect, Characterize, and Remove Motion Artifact in Resting State fMRI.” NeuroImage 84 (Supplement C): 320–41. https://doi.org/10.1016/j.neuroimage.2013.08.048.

Satterthwaite, Theodore D., Mark A. Elliott, Raphael T. Gerraty, Kosha Ruparel, James Loughead, Monica E. Calkins, Simon B. Eickhoff, et al. 2013. “An improved framework for confound regression and filtering for control of motion artifact in the preprocessing of resting-state functional connectivity data.” NeuroImage 64 (1): 240–56. https://doi.org/10.1016/j.neuroimage.2012.08.052.

Tustison, N. J., B. B. Avants, P. A. Cook, Y. Zheng, A. Egan, P. A. Yushkevich, and J. C. Gee. 2010. “N4ITK: Improved N3 Bias Correction.” IEEE Transactions on Medical Imaging 29 (6): 1310–20. https://doi.org/10.1109/TMI.2010.2046908.

Zhang, Y., M. Brady, and S. Smith. 2001. “Segmentation of Brain MR Images Through a Hidden Markov Random Field Model and the Expectation-Maximization Algorithm.” IEEE Transactions on Medical Imaging 20 (1): 45–57. https://doi.org/10.1109/42.906424.
